# Supplementary material for: Structural and Functional Comparison of Salmonella Flagellar Filaments Composed of FljB and FliC
Source: Biomolecules. 2020 Feb 6;10(2):246. doi: 10.3390/biom10020246 (PMC7072241; doi:10.3390/biom10020246)
Supplement: Supplementary file 1 [file biomolecules-10-00246-s001.pdf]

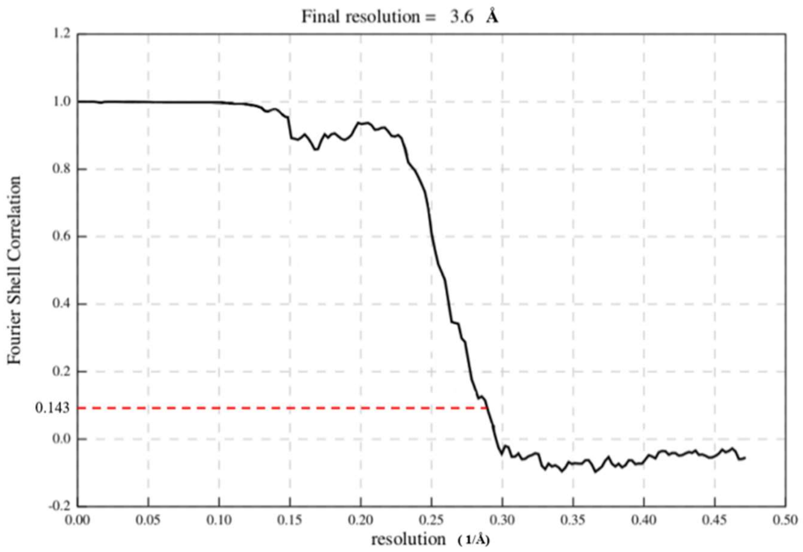

Figure 1. Fourier shell correlation of the 3D reconstruction.

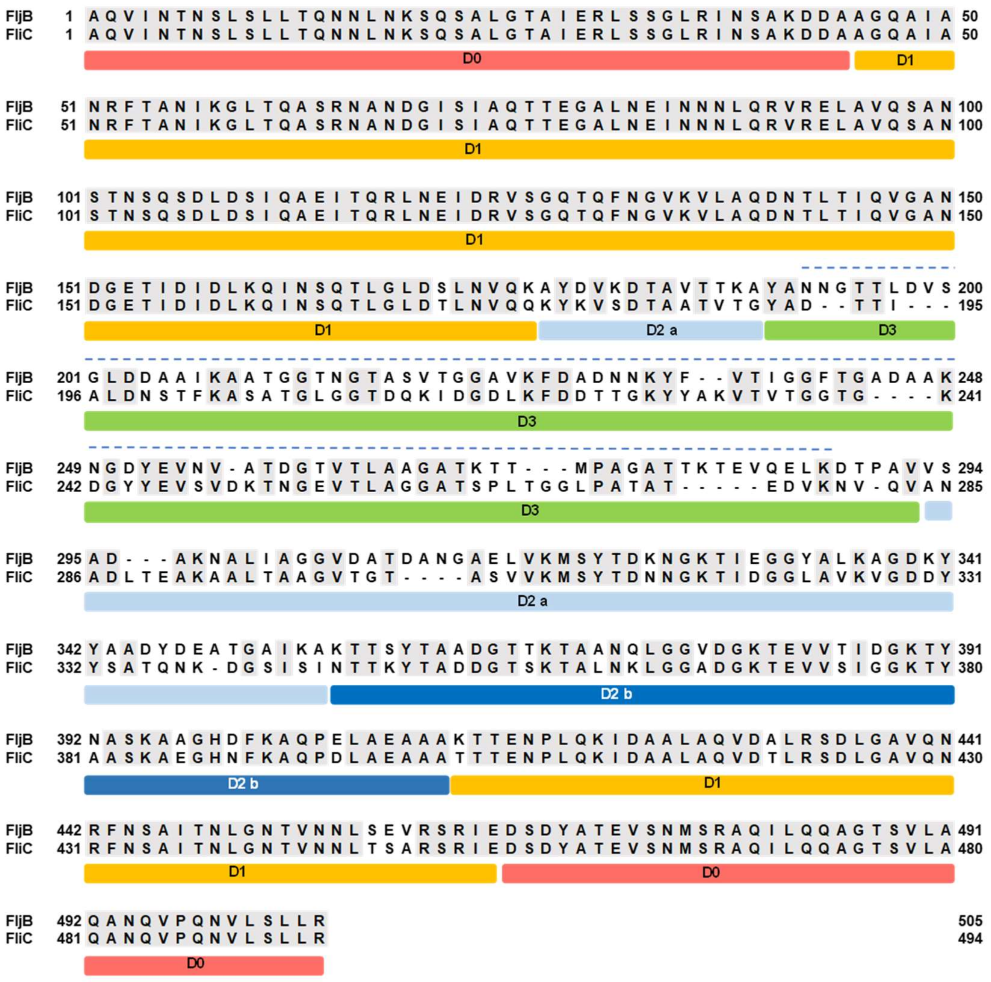

Figure 2. Amino acid sequence alignment of FljB and FliC. The amino acid sequence of FljB and FliC shows 76% identity and 1.4×10^{-66} *E*-value. Conserved residues are shaded in gray. The regions of domains are indicated by bars and labels in different colors underneath the sequence (D0, red; D1, orange; D2a, right blue; D2b, dark blue; D3, green). The dashed line above the sequence indicates the missing regions of the FljB model (6JY0).

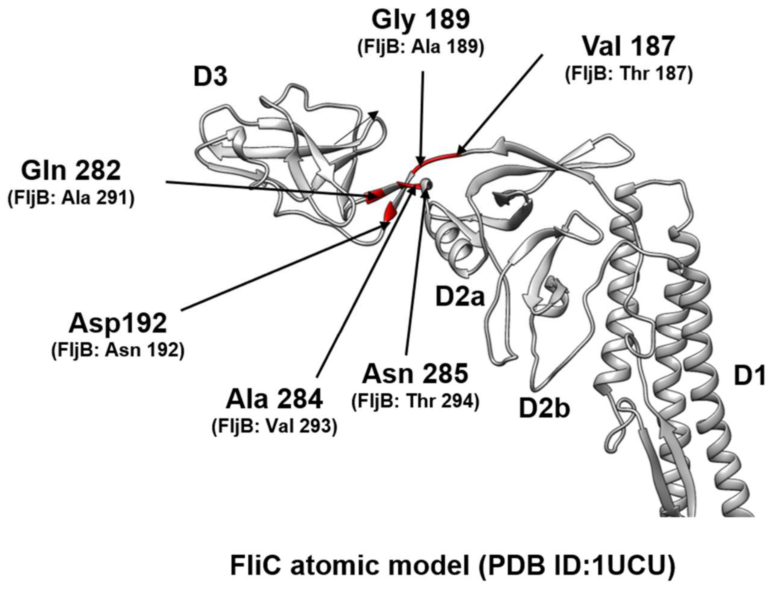

Figure 3. Differences in residue numbers and types between FliC and FljB mapped on the FliC model. Differences are shown for residues 187 – 192 and 282 – 285 of FliC and residues 187 – 192 and 291 – 294 of FljB. The FliC chain is colored red for those residues that are different from FljB.

Table S1. Summary of cryoEM data collection and image analysis

**Data collection**	
Microscope	Titan Krios
Camera	Falcon Ⅱ
Data acquisition	Auto / EPU
Voltage (kV)	300
Magnification	75,000
Pixel size (Å)	1.06
Defocus range (μm)	0.2 -1.9
Frame rate (sec/frame)	0.1
Total exposure time (sec)	2
Number of frames	7
Dose rate (e^-/Å^2/frame)	10.3
Total dose (e^-/Å)	72.1
**Particle statistics**	
Number of micrographs	2,319
Number of picked segments	197,442 (90% overlap)
Number of segments (final)	114,110
Resolution (Å)	3.6
FSC threshold	0.143
**Ramachandran plot**	
Favored (%)	94
Allowed (%)	6
Outliers (%)	1
